# A Simple Preoperative Blood Count to Stratify Prognosis in Isocitrate Dehydrogenase-Wildtype Glioblastoma Patients Treated with Radiotherapy plus Concomitant and Adjuvant Temozolomide

**DOI:** 10.3390/cancers13225778

**Published:** 2021-11-18

**Authors:** Anne Clavreul, Jean-Michel Lemée, Gwénaëlle Soulard, Audrey Rousseau, Philippe Menei

**Affiliations:** 1 Université d’Angers, CHU d’Angers, CRCINA, F-49000 Angers, France; JMLemee@chu-angers.fr (J.-M.L.); aurousseau@chu-angers.fr (A.R.); phmenei@chu-angers.fr (P.M.); 2 Département de Neurochirurgie, CHU Angers, F-49933 Angers, France; Gwsoulard@chu-angers.fr; 3 Département de Pathologie Cellulaire et Tissulaire, CHU Angers, F-49933 Angers, France

**Keywords:** glioblastoma, hematological markers, survival, prognosis

## Abstract

**Simple Summary:**

Glioblastoma (GB) is the most common primary malignant brain tumor in adulthood. The median survival of patients is approximately 15 months after the standard therapy including safe maximal resection followed by radiotherapy plus concomitant and adjuvant temozolomide. However, the survival times of GB patients undergoing this treatment are heterogeneous, with a small fraction living even beyond 36 months. The identification of a reliable and simple method for predicting whether patients will be short- or long-term survivors could assist in shaping individualized posttreatment surveillance. We show here that a simple, low-cost, relatively innocuous blood test before surgery can predict the survival outcomes of patients with isocitrate dehydrogenase (IDH)-wildtype GB treated with the standard therapy.

**Abstract:**

Purpose: The survival times of glioblastoma (GB) patients after the standard therapy including safe maximal resection followed by radiotherapy plus concomitant and adjuvant temozolomide are heterogeneous. In order to define a simple, reliable method for predicting whether patients with isocitrate dehydrogenase (IDH)-wildtype GB treated with the standard therapy will be short- or long-term survivors, we analyzed the correlation of preoperative blood counts and their combined forms with progression-free survival (PFS) and overall survival (OS) in these patients. Methods: Eighty-five patients with primary IDH-wildtype GB treated with the standard therapy between 2012 and 2019 were analyzed retrospectively. Cox proportional hazards models and Kaplan–Meier analysis were used to investigate the survival function of preoperative hematological parameters. Results: Preoperative high neutrophil-to-lymphocyte ratio (NLR, >2.42), high platelet count (>236 × 10^9^/L), and low red blood cell (RBC) count (≤4.59 × 10^12^/L) were independent prognostic factors for poorer OS (*p* = 0.030, *p* = 0.030, and *p* = 0.004, respectively). Moreover, a high NLR was an independent prognostic factor for shorter PFS (*p* = 0.010). We also found that, like NLR, preoperative high derived NLR (dNLR, >1.89) was of poor prognostic value for both PFS (*p* = 0.002) and OS (*p* = 0.033). A significant correlation was observed between NLR and dNLR (r = 0.88, *p* < 0.001), which had a similar prognostic power for OS (NLR: AUC = 0.58; 95% CI: [0.48; 0.68]; dNLR: AUC = 0.62; 95% CI: [0.51; 0.72]). Two scores, one based on preoperative platelet and RBC counts plus NLR and the other on preoperative platelet and RBC counts plus dNLR, were found to be independent prognostic factors for PFS (*p* = 0.006 and *p* = 0.002, respectively) and OS (*p* < 0.001 for both scores). Conclusion: Cheap, routinely ordered, preoperative assessments of blood markers, such as NLR, dNLR, RBC, and platelet counts, can predict the survival outcomes of patients with IDH-wildtype GB treated with the standard therapy.

## 1. Introduction

Glioblastoma (GB) is the most common primary malignant brain tumor in adulthood. Despite the standard therapy based on safe maximal resection followed by radiotherapy plus concomitant and adjuvant temozolomide (TMZ, Stupp protocol), the median survival of GB patients is only about 15 months [1]. However, survival is highly heterogeneous in GB patients, with rates of 18% at two years, 11% at three years and 4% at five years [2]. Efforts are currently being made to identify prognostic parameters for short or long survival in these patients.

Many patient characteristics, including age, sex, performance status, and tumor site, have been identified as potential prognostic factors [3,4]. Furthermore, molecular markers, such as isocitrate dehydrogenase (IDH) mutations and O^6^-methylguanine-DNA-methyltransferase (MGMT) hypermethylation are increasingly being used as predictors of prognosis and therapeutic response in GB patients [5,6,7]. There is growing evidence to suggest that preoperative hematological biomarkers, which reflect the tumor microenvironment to some extent, could be used as diagnostic and prognostic markers in several cancers, including GB [8,9]. For example, previous studies have indicated that neutrophil-to-lymphocyte ratio (NLR), platelet-to-lymphocyte ratio (PLR), and lymphocyte-to-monocyte ratio (LMR) are associated with the clinical outcomes of GB [9,10,11].

The classification of GB based on such preoperative hematological biomarkers could potentially improve the stratification of patient prognosis and would require no more than a cheap, simple, relatively innocuous blood test. However, many of the previous studies analyzed GB as a single entity, without distinguishing between IDH-wildtype and IDH-mutant GB. It is now widely accepted that IDH-wildtype and IDH-mutant GB are fundamentally different, with distinctive methylation and gene expression profiles [12]. Most IDH-mutant GB are secondary GB developing from low-grade gliomas and are less aggressive than de novo or primary IDH-wildtype GB [12]. It has been shown that *IDH1* mutation is associated with lower levels of chronic inflammation, potentially accounting for the better prognosis of patients with such mutations [13,14,15]. In its new classification of central nervous system tumors, the World Health Organization (WHO) refers to IDH-mutant GB as grade 4 mutated IDH astrocytoma to distinguish more clearly between this entity and non-mutated IDH GB [16,17].

Here, we analyzed the influence of various preoperative hematological parameters, such as red blood cell (RBC), white blood cell (WBC), neutrophil, lymphocyte, and platelet counts, and several combinations of these factors, such as NLR, derived NLR (dNLR), PLR, LMR, the systemic immune-inflammation index (SII), and the systemic inflammation response index (SIRI), on overall survival (OS) and progression-free survival (PFS) in patients with IDH-wildtype GB treated with the standard therapy.

## 2. Patients and Methods

### 2.1. Patients

This retrospective study included patients who were newly diagnosed with IDH-wildtype GB between January 2012 and December 2020 at Angers University Hospital. The following inclusion criteria were used: (1) patient aged ≥ 18 years, (2) newly diagnosed unilateral supratentorial GB, (3) GB without immunohistochemical staining for IDH1-R132H, (4) tumor resected, (5) no intraoperative chemotherapy, (6) complete data for routine blood tests before surgery, and (7) first-line treatment with complete concurrent chemoradiotherapy according to the Stupp protocol [1]. The number of cycles of subsequent adjuvant chemotherapy with oral TMZ depended on tolerance and radiological response. Patients with acute infection, chronic active inflammatory disease, autoimmune disease, hematological disorders, or other tumors were excluded. Patients on corticosteroids before the preoperative blood test were also excluded. Using these criteria, 85 patients were included. For this retrospective study, French legislation required only authorization from the French National Data Protection Authority (CNIL; authorization no. ar19-0053v0/no. 1476342) and the non-objection of the patients to the use of their personal data.

### 2.2. Data Collection

Baseline characteristics, such as age, sex, preoperative Karnofsky performance score (KPS), tumor location, blood data, extent of resection (EOR), and Stupp protocol regimen, were collected from medical records. The preoperative hematological parameters extracted from blood data included absolute counts of RBC, WBC, neutrophils, lymphocytes, monocytes, and platelets. These absolute counts were then used for the calculation of several combined variables: NLR = neutrophil count/lymphocyte count, PLR = platelet count/lymphocyte count, LMR = lymphocyte count/monocyte count, dNLR = neutrophil count/(total WBC count—neutrophil count), SII = (platelet count × neutrophil count)/lymphocyte count, and SIRI = (neutrophil count × monocyte count)/lymphocyte count. EOR was recorded by the surgeon performing the operation or was determined from a postoperative MRI scan performed within 48 h of surgery, by a neuroradiologist. EOR was classified as gross total (GTR; 100%), subtotal (STR; ≥90%), or partial (PR; <90%). OS was defined as the time from initial surgery to death. PFS was defined as time from first surgery to radiological progression according to the RANO criteria [18].

### 2.3. Statistical Analysis

Pearson’s chi-squared test was used to evaluate the relationship between variables. Univariate Cox regression analysis was performed with the clinical and hematological covariates of all patients to screen for factors associated with PFS and OS. All covariates were analyzed as dichotomous variables. The optimal cutoff for continuous hematological variables was determined using the maximally selected rank statistics from the ‘maxstat’ R package. *p*-values were adjusted by the Bonferroni method for multiple testing. Variables with raw *p*-values < 0.05 in univariate analysis were included in multivariate Cox regression analysis unless they were redundant or correlated with each other. Moreover, demographic variables, displaying an interaction with hematological variables included in the model, were forced into the model, regardless of their significance. The global statistical significance of the Cox model was checked in three alternative tests (likelihood ratio, Wald, and log-rank). The Cox model was also tested by two types of diagnostics: Schoenfeld residuals to verify the assumption of proportional hazards and the determination of dfbeta values for the investigation of influential outliers. Survival curves were plotted according to the Kaplan–Meier method and compared in log-rank tests. A receiver operating characteristic (ROC) curve was also generated and the area under the curve (AUC) was calculated to evaluate the prognostic power for OS of the hematological markers. Statistical analyses were performed with R software (version 4.1.0). Values of *p* < 0.05 were considered statistically significant.

## 3. Results

### 3.1. Patient Characteristics

The baseline characteristics of the 85 selected IDH-wildtype GB patients are shown in Table 1. Mean age at diagnosis was 61.5 ± 8.8 years and 65 patients (76%) were male. Seventy-one patients (84%) had a KPS score > 80% before surgery. The GB was in the left hemisphere in 42 patients (49%) and the right hemisphere in 43 patients (51%). GB was unilobar in 46 patients (54%) and multilobar in 39 patients (46%). The EOR was complete in 46 patients (54%). All patients received concurrent chemoradiotherapy according to the Stupp protocol. However, 70 patients (82%) had no more than six cycles of adjuvant TMZ and 15 patients (18%) had more than six cycles. The 85 GB patients had a median PFS of 7.4 months (95% CI: [6.7; 8.7]) and a median OS of 17.7 months (95% CI: [14.5; 21.6]) (Table 1).

### 3.2. Univariate and Multivariate Analyses

Six variables were associated with a shorter PFS in univariate analysis: short TMZ consolidation treatment (*p* < 0.001), low RBC count (*p* = 0.032), high WBC count (*p* = 0.029), high NLR (*p* = 0.027), high dNLR (*p* = 0.002), and high SIRI (*p* = 0.045) (Table 2). Twelve variables were associated with a shorter OS in univariate analysis: low KPS (*p* = 0.009), multilobar location (*p* = 0.022), short TMZ consolidation treatment (*p* < 0.001), low RBC count (*p* = 0.002), high WBC count (*p* = 0.004), high neutrophil count (*p* = 0.004), high lymphocyte count (*p* = 0.025), high platelet count (*p* = 0.046), high NLR (*p* = 0.007), high dNLR (*p* = 0.002), high SII (*p* = 0.034), and high SIRI (*p* = 0.003) (Table 2).

Pearson’s chi-squared test showed that NLR, dNLR, and SII were strongly correlated and that this correlation was strongest between NLR and dNLR (r = 0.88, *p* < 0.001; Table S1). Prognostic power for OS was similar for NLR (AUC = 0.58; 95% CI: [0.48; 0.68]), dNLR (AUC = 0.62; 95% CI: [0.51; 0.72]) and SII (AUC = 0.56; 95% CI: [0.47; 0.66]). We chose to include NLR and dNLR in the multivariate Cox regression analysis and to generate two separate models for these two variables because they were more strongly associated with PFS and OS than SII (Table 2). Neither NLR nor dNLR was associated with platelet or RBC counts (Table S1). Leukocyte, neutrophil, and lymphocyte counts and SIRI, all of which were weakly or moderately associated with NLR or dNLR, were excluded from the Cox model to avoid redundancy (Table S1). The demographic variable “sex”, for which an association was found with RBC count in our cohort study (*p* = 0.039) was forced into the model. We identified three variables as independently associated with shorter PFS in multivariate analysis for the model including NLR: short TMZ consolidation treatment (*p* < 0.001), low RBC count (*p* = 0.048), and high NLR (*p* = 0.010) (Table 3). Following the replacement of NLR with dNLR, the multivariate analysis also showed that dNLR was an independent prognostic factor for PFS (*p* = 0.002), but the only other significant variable in this model was TMZ consolidation treatment (*p* < 0.001) (Table 3). We identified five variables as independently associated with shorter OS in multivariate analysis for the model including NLR: male gender (*p* = 0.028), short TMZ consolidation treatment (*p* < 0.001), low RBC count (*p* = 0.004), high platelet count (*p* = 0.030), and high NLR (*p* = 0.030) (Table 3). When NLR was replaced with dNLR in the model, the multivariate analysis also showed that gender (*p* = 0.023), TMZ consolidation treatment (*p* < 0.001), RBC count (*p* = 0.003), platelet count (*p* = 0.041), and dNLR (*p* = 0.033) were independent prognostic factors for OS (Table 3). No association between the number of cycles of TMZ maintenance and NLR (*p* = 0.176), platelet (*p* = 0.668), or RBC counts (*p* = 0.504) was observed, but there was a slight negative association between TMZ consolidation treatment and dNLR (*p* = 0.050).

### 3.3. Survival Analysis of Independent Prognostic Hematological Markers

**Figure 1:**
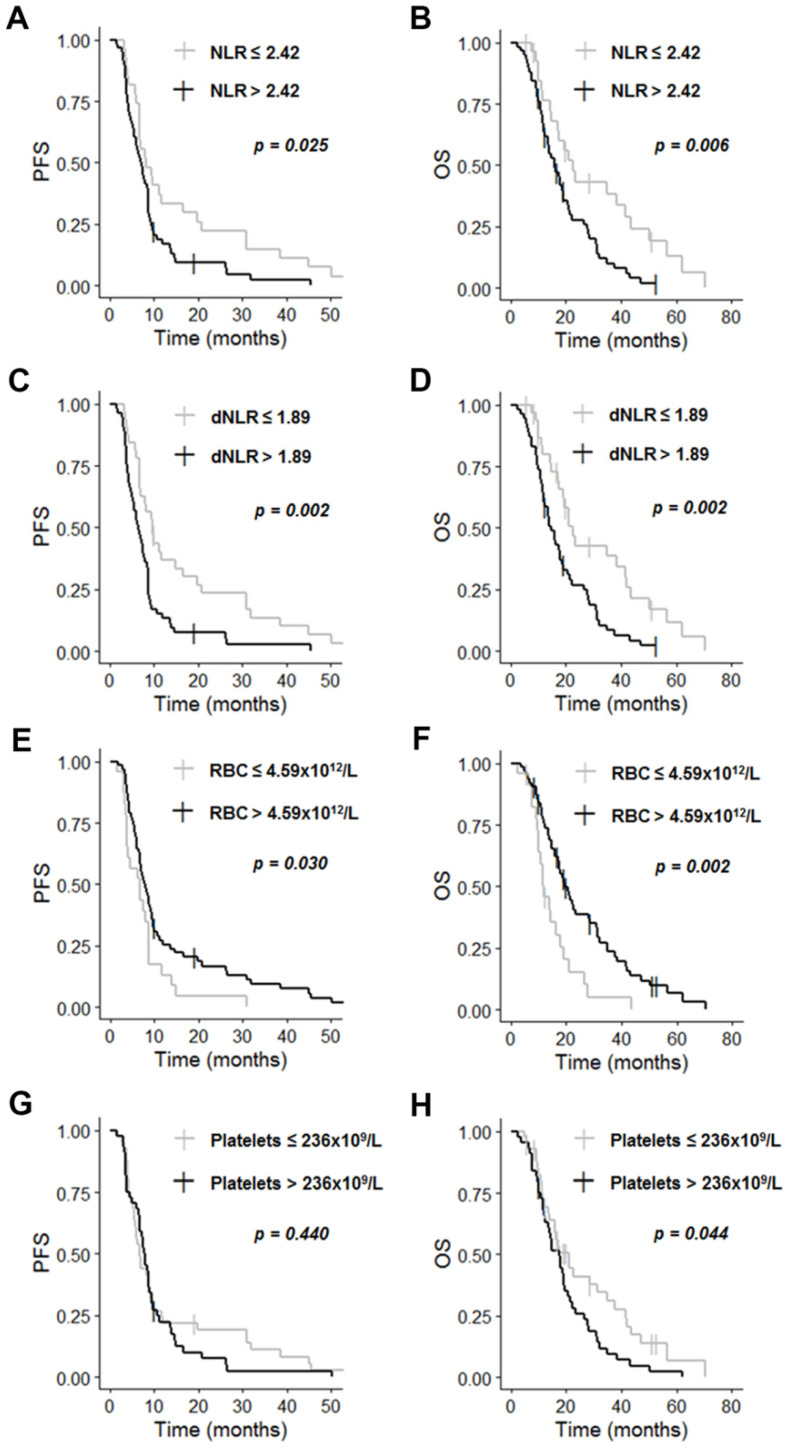
Kaplan–Meier curves for survival stratified by NLR ((**A**) PFS; (**B**) OS), dNLR ((**C**) PFS, (**D**) OS), RBC ((**E**) PFS; (**F**) OS) and platelets ((**G**) PFS; (**H**) OS). Abbreviations: dNLR, derived neutrophil-to-lymphocyte ratio, NLR, neutrophil-to-lymphocyte ratio; OS, overall survival; PFS, progression-free survival; RBC, red blood cells.

As shown in Figure 1, patients with high NLR, high dNLR, or low RBC count had a significantly poorer PFS and OS. Median PFS was 7.1 months (95% CI: [5.6; 8.7]) for patients with NLR > 2.42, and 8.3 months (95% CI: [6.8; 19.8]) for patients with NLR ≤ 2.42 (*p* = 0.025, Figure 1A). Median OS was 16.0 months (95% CI: [13.6; 20.7]) for patients with NLR > 2.42, and 22.7 months (95% CI: [17.1; 50.0]) for patients with NLR ≤ 2.42 (*p* = 0.006, Figure 1B). Median PFS was 6.4 months (95% CI: [5.3; 8.5]) for patients with dNLR > 1.89, and 9.7 months (95% CI: [7.0; 19.8]) for patients with dNLR ≤ 1.89 (*p* = 0.002, Figure 1C). Median OS was 14.8 months (95% CI: [12.1; 19.1]) for patients with dNLR > 1.89 and 22.7 months (95% CI: [19.0; 43.4]) for patients with dNLR ≤ 1.89 (*p* = 0.002, Figure 1D). Median PFS was 6.7 months (95% CI: [3.7; 8.7]) for patients with RBC counts ≤ 4.59 × 10^12^/L and 7.8 months (95% CI: [6.8; 9.5]) for patients with RBC counts > 4.59 × 10^12^/L (*p* = 0.030, Figure 1E). Median OS was 12.1 months (95% CI: [10.1; 19.0]) for patients with RBC counts ≤ 4.59 × 10^12^/L and 19.4 months (95% CI: [17.1; 28.4]) for patients with RBC counts > 4.59 × 10^12^/L (*p* = 0.002, Figure 1F). The OS of patients with a high platelet count was also significantly lower than that of patients with a low platelet count. Median OS was 17.5 months (95% CI: [13.6; 21.6]) for patients with platelet counts > 236 × 10^9^/L and 20.8 months (95% CI: [15.8; 37.7]) for patients with platelet counts ≤ 236 × 10^9^/L (*p* = 0.044, Figure 1H). There was no significant difference in PFS between the two groups. Median PFS was 7.8 months (95% CI: [6.7; 9.3]) for patients with platelet counts > 236 × 10^9^/L and 6.8 months (95% CI: [5.8; 9.2]) for patients with platelet counts ≤ 236 × 10^9^/L (*p* = 0.440, Figure 1G).

### 3.4. A Scoring System Based on Preoperative Platelet and RBC Counts plus NLR or dNLR

**Figure 2:**
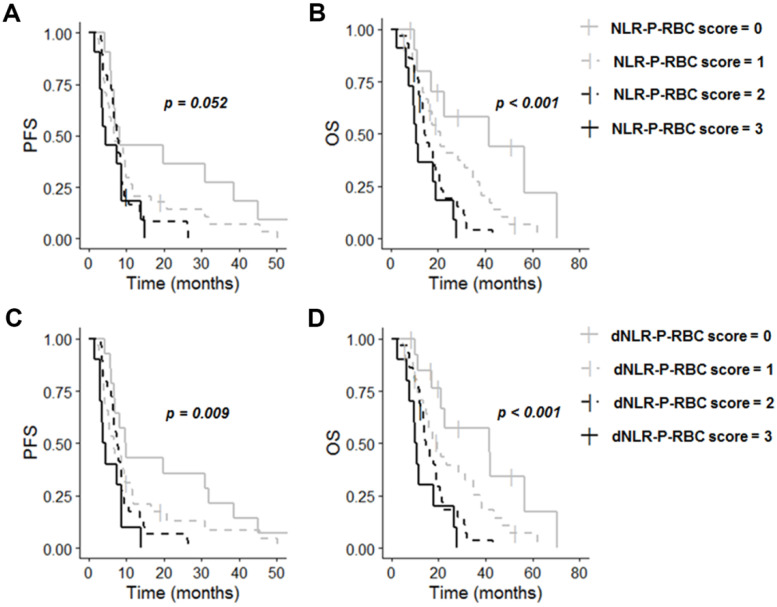
Kaplan–Meier curves for survival stratified by NLR-P-RBC score ((**A**) PFS; (**B**) OS) and dNLR-P-RBC score ((**C**) PFS; (**D**) OS). Abbreviations: dNLR-P-RBC, derived neutrophil-to-lymphocyte ratio-platelet count-red blood cell count; NLR-P-RBC, neutrophil-to-lymphocyte ratio-platelet count-red blood cell count; OS, overall survival; PFS, progression-free survival.

We established a score based on preoperative NLR, platelet, and RBC counts. This score, NLR-P-RBC, was calculated as follows: score = 3, patients with three abnormalities (*n* = 11) (high NLR, high platelet count, and low RBC count), score = 2, patients with two of these abnormalities (*n* = 29), score = 1, patients with only one abnormality (*n* = 34), and score = 0, patients without abnormalities (*n* = 11). NLR-P-RBC score was significantly associated with PFS (OR = 1.44; 95% CI: [1.11; 1.87]; *p* = 0.006; Adj *p* = 0.126) and OS (OR = 1.86; 95% CI: [1.40; 2.48]; *p* < 0.001; Adj *p* < 0.001) in univariate analysis. This score remained an independent prognostic factor for PFS and OS in multivariate analysis (Table 3). Median PFS in patients with three abnormalities for the preoperative blood test (score = 3) was shorter than that in patients with NLR-P-RBC score of 2, 1, or 0 (*p* = 0.052). Median PFS was 8.3 months (95% CI: [6.8; NA]) for patients with an NLR-P-RBC score of 0, 6.9 months (95% CI: [5.6; 9.9]) for patients with an NLR-P-RBC score of 1, 7.6 months (95% CI: [6.7; 8.8]) for patients with an NLR-P-RBC score of 2 and 4.4 months (95% CI: [3.4; NA]) for patients with an NLR-P-RBC score of 3 (Figure 2A). Median OS in patients with an NLR-P-RBC score of 3 for the preoperative blood test was also shorter than that of patients with an NLR-P-RBC score of 2, 1, or 0 (*p* < 0.001). Median OS was 41.7 months (95% CI: [17.06; NA]) for patients with an NLR-P-RBC score of 0, 20.8 months (95% CI: [15.8; 34.8]) for patients with an NLR-P-RBC score of 1, 14.2 months (95% CI: [12.3; 21.6]) for patients with an NLR-P-RBC score of 2 and 10.8 months (95% CI: [9.8; NA]) for patients with an NLR-P-RBC score of 3 (Figure 2B).

When NLR was replaced with dNLR in the scoring system (score = 3 (*n* = 10); score = 2 (*n* = 29); score = 1 (*n* = 32); score = 0 (*n* = 14)), univariate analysis showed that dNLR-P-RBC score was also significantly associated with PFS (OR = 1.53; 95% CI: [1.18; 1.99]; *p* = 0.001; Adj *p* = 0.021) and OS (OR = 1.87; 95% CI: [1.41; 2.48]; *p* < 0.001; Adj *p* < 0.001). This score also remained an independent prognostic factor for PFS and OS in multivariate analysis (Table 3). Median PFS in patients with a dNLR-P-RBC score of 3 for the preoperative blood test was shorter than that in patients with dNLR-P-RBC score of 2, 1, or 0 (*p* = 0.009). Median PFS was 9.8 months (95% CI: [7.0; 45.0]) for patients with a dNLR-P-RBC score of 0, 6.7 months (95% CI: [5.3; 11.2]) for patients with a dNLR-P-RBC score of 1, 7.6 months (95% CI: [6.7; 8.8]) for patients with a dNLR-P-RBC score of 2 and 4.0 months (95% CI: [3.1; NA]) for patients with a dNLR-P-RBC score of 3 (Figure 2C). Median OS in patients with dNLR-P-RBC score of 3 was also shorter than that of patients with a dNLR-P-RBC score of 2, 1 or 0 (*p* < 0.001). Median OS was 41.7 months (95% CI: [21.0; NA]) for patients with a dNLR-P-RBC score of 0, 19.4 months (95% CI: [14.5; 34.8]) for patients with a dNLR-P-RBC score of 1, 14.8 months (95% CI: [13.6; 20.7]) for patients with a dNLR-P-RBC score of 2, and 10.4 months (95% CI: [7.4; NA]) for patients with a dNLR-P-RBC score of 3 (Figure 2D).

## 4. Discussion

Survival time is heterogeneous for GB patients undergoing the standard therapy, with a small fraction surviving even beyond 36 months [1,2]. The definition of a simple, reliable method for predicting whether patients are likely to be long- or short-term survivors would be beneficial, as it would make it possible to adapt individualized post-treatment surveillance. In this study, we retrospectively evaluated the prognostic value of preoperative blood counts and their combined forms in 85 newly diagnosed IDH-wildtype GB patients treated with the standard therapy. All patients received concurrent radiation therapy and TMZ chemotherapy as first-line treatment, but the TMZ consolidation treatment varied in these patients, with only 15 patients (18%) receiving more than six cycles of oral TMZ. We found that the duration of TMZ consolidation treatment was an independent predictive factor for PFS and OS. This is not particularly surprising as the number of cycles is dependent on tolerance and radiological response. The survival benefits of extended adjuvant TMZ in newly diagnosed GB cases have already been highlighted in several other studies [19,20,21]. Consistent with published findings, we observed an association of sex with OS in multivariate analysis in this selected cohort of 85 GB patients [4]. However, age, KPS and EOR were not found to be independent factors associated with OS in this cohort. The small number of patients and the lack of quantitative MRI assessment of volume for EOR evaluation may account for this discrepancy.

We found that preoperative NLR, dNLR, platelet count, and RBC count were independent prognostic factors for OS, with high NLR (>2.42), high dNLR (>1.89), high platelet count (>236 × 10^9^/L) and low RBC count (≤4.59 × 10^12^/L) being associated with poor outcome. Other hematological markers related to NLR, dNLR, or platelets, including WBC count, lymphocyte count, neutrophil count, SII, and SIRI were also associated with OS in univariate analysis. Preoperative NLR > 2.42 and dNLR > 1.89 can also be considered to be independent prognostic factors associated with a poorer PFS. Monocyte counts, PLR, and LMR had no significant impact on PFS or OS in univariate analysis.

NLR has been reported to be of prognostic value for various cancers, including GB [8,9]. Our findings are consistent with those of several studies showing an association, in univariate analysis, of higher preoperative NLR values (cutoffs ranging from 1.7–7) with poor OS in GB patients. At least 11 retrospective studies have been completed in different countries, with total populations of 84 (cutoff > 4, Ireland) [22], 152 (cutoff ≥ 4, China) [23], 141 (cutoff > 4, China) [24], 90 (cutoff ≥ 5, Turkey) [25], 117 (cutoff > 7, Portugal) [26], 105 (cutoff ≥ 4, China) [27], 192 (cutoff > 2.7, China) [28], 124 (cutoff ≥ 4, Italy) [29], 497 (cutoff > 4, the Netherlands) [30], 129 (continuous variable, India) [31], and 194 (cutoff > 1.706, China) [32] GB patients, and preliminary results are available for one prospective study (51 patients, cutoff > 4.73, Greece) [33]. In accordance with our study, all but one [30] of these studies reported that higher preoperative NLR values remained an independent prognostic factor for poor outcome in multivariate analysis. Mason et al. [34] also confirmed that a high NLR just before or during focal radiotherapy and concomitant TMZ was associated with a poorer prognosis. Five studies reported no significant correlation between NLR and OS in uni- and multivariate analyses with total populations of 84 (cutoff ≥ 4, China) [35], 80 (cutoff > 4, Turkey) [36], 89 (cutoff > 2.5, 3 or 4, Israel) [37], and 87 (cutoff > 5.07, Australia) [38] GB patients. These discrepancies may reflect differences in hematological instruments, methods for determining cutoff values, sample size, surgical options, and adjuvant treatment regimens. We found that a preoperative NLR > 2.42 was correlated with a shorter PFS in uni- and multivariate analyses. The prognostic value of preoperative NLR has been less frequently analyzed for PFS than for OS. We identified five studies in which such analyses were performed and, contrary to our findings, four of these studies reported no correlation between a higher preoperative NLR and poor PFS [29,36,37,38]. In one study, this correlation was found in patients with GB but was not confirmed when the analysis was limited to the subgroup of patients who completed the Stupp protocol in which a high preoperative NLR was correlated with a shorter OS [26]. Despite these conflicting data, we found that, like NLR, preoperative dNLR was of prognostic value for both PFS and OS. dNLR, which is based on WBC and neutrophil counts, was initially defined by Proctor et al. [39]. The diagnostic value of preoperative dNLR for predicting glioma grade has been highlighted [15,40]. Our findings are consistent with those of Madhugiri et al. [31] showing an association of higher dNLR with shorter OS in GB patients. We observed a significant correlation between NLR and dNLR, which were of similar prognostic value for OS. Thus, as for other cancers, dNLR may be used as an alternative to NLR for predicting survival in GB patients [8,39]. Many studies have shown that circulating neutrophil levels are a major determinant of immunosuppression, progression, and treatment resistance in GB [41,42,43,44,45,46,47]. Glioma grade is positively correlated with the levels of circulating and tumor-infiltrating neutrophils [42,47,48]. Moreover, a positive correlation has been found between elevated peripheral blood NLR levels and high levels of tumor neutrophil infiltration/low levels of CD3-positive T-cell infiltration in GB [23,48]. Neutrophils are recruited to the GB site by many chemotactic agents, including IL-8 or chemokine ligand 8 and macrophage migration inhibitory factor [41]. The underlying mechanisms by which tumor-infiltrating neutrophils promote the progression of GB and other cancers in general remain to be revealed, and their study is complicated by the multiplicity of plasticity phenotypes and functionalities [41,49,50]. Liang et al. [42] showed that neutrophils enhance the proliferation of glioma stem cells (GSCs) by upregulating S100A4 expression, leading to tumor growth and resistance to anti-vascular endothelial growth factor (VEGF) therapy in GB. Zha et al. [47] have provided evidence to suggest that neutrophil extracellular traps (NETs) secreted by tumor-infiltrating neutrophils promote GB cell proliferation, migration, and invasion. NETs are complex extracellular structures composed of chromatin and specific proteins, including histones, granule enzyme myeloperoxidase, cathepsin G, leukocyte proteinase 3, and lysozyme C [51]. Yee et al. [46] showed that neutrophil-induced ferroptosis promotes necrosis in GB and is associated with mesenchymal transition and positively correlated with tumor aggressiveness in human GB. Tumor-associated neutrophils appear to play a crucial role in GB progression, but their use as treatment targets is likely to be challenging. Neutrophils are crucial mediators of host defense against infection, and their depletion may result in dangerous levels of immunosuppression. Liang et al. [42] found that downregulating S100A4 expression in GSCs inhibited neutrophil-promoted tumor progression regardless of the degree of neutrophil infiltration. The targeting of this specific neutrophil-activated regulator on tumor cells provides a possible alternative treatment strategy.

In addition to the prognostic value of preoperative NLR or dNLR, we found that a high preoperative platelet count (>236 × 10^9^/L) was associated with a poorer OS in uni- and multivariate analyses. Three studies reported relationships between preoperative thrombocytosis and poorer OS in univariate analysis on 153 (cutoff > 400 × 10^9^/L, Germany) [52], 84 (cutoff ≥ 151 × 10^9^/L, China) [35], and 124 (cutoff > 350 × 10^9^/L, Italy) [29] GB patients. Moreover, preoperative thrombocytosis remained an independent prognostic indicator of poor outcome in multivariate analysis in two of these studies [29,52]. Preoperative thrombocytosis has also been reported to be of prognostic value in other cancers, being closely associated with poor outcomes in colorectal cancer, nonsmall cell lung cancer, and ovarian carcinoma [53,54,55]. Nevertheless, caution is required in the use of circulating platelet count as a prognostic marker in GB. Three studies reported no significant prognostic value of platelet count for GB with total population of 140 (continuous variable, Portugal) [26], 107 (continuous variable, Turkey) [56], and 497 (cutoff > 450 × 10^9^/L, the Netherlands) [30] GB patients. Furthermore, in this and other studies, PLR, another platelet parameter, was not found to be of prognostic value [25,28,31,32,35,36]. To our knowledge, two studies have reported an influence of PLR on survival in univariate analysis [23,29], and one study found PLR to be an independent prognostic factor, low PLR being associated with a better prognosis [24]. The contribution of platelets to tumor development, invasiveness, malignancy, and metastasis is well recognized in solid tumors outside the central nervous system, but the contribution of these cells to GB pathophysiology remains unclear [57]. Brockmann et al. [52] showed preoperative thrombocytosis to be a prognostic factor associated with shorter survival time in patients with GB, but they found no correlation between preoperative platelet count and proliferative activity and vessel density in tumor samples from GB patients [58]. Recent studies have reported a higher activation status of circulating platelets in patients diagnosed with primary GB, and these circulating activated platelets are able to affect the GB microenvironment by supplying oncopromoter and proangiogenic factors such as von Willebrand factor, VEGF, and sphingosine-1-phosphate [59,60,61]. Mean platelet volume (MPV) and MPV/platelet count ratio, two markers of platelet activity, may be independent predictors of survival in patients with GB [56,62]. All these data demonstrate that, although platelets appear to contribute to GB progression, additional studies are required to gain greater insight into the roles of circulating platelet number and activation levels as possible prognostic markers in GB.

We also observed that a low preoperative RBC count (≤4.59 × 10^12^/L) was associated with poorer OS in uni- and multivariate analyses. RBC parameters are known to be associated with cancer outcomes, with preoperative anemia associated with poor survival outcomes in patients with various cancers, including renal cell carcinoma, gastric cancer, colorectal cancer, and lung cancer [63,64,65,66]. In the context of GB, few studies have considered this topic, and different RBC parameters have been studied. Some studies provided evidence that low preoperative hemoglobin levels are associated with a poor prognosis [67,68], but others provided less support for this notion [29,30,35,69]. In our study, unlike RBC count, hemoglobin level was not predictive of survival in patients with GB (data not shown). Few studies have analyzed the prognostic value of preoperative RBC count in GB patients. Liang et al. [69], in addition to evaluating the prognostic impact of preoperative RBC count, also analyzed several other RBC parameters, including mean cell volume, hemoglobin and mean corpuscular hemoglobin levels, hematocrit, and RBC distribution width (RDW). They showed that only RDW, reflecting the heterogeneity of circulating RBC size, was associated with patient OS in uni- and multivariate analyses. Kaisman-Elbaz et al. [67] also showed that RDW was an independent prognostic factor in patients with GB; a high RDW was associated with a poor OS in these patients. These data highlight the association of RBC parameters with GB, although the roles of these parameters in GB pathophysiology remain mostly unknown. Published data have highlighted the association between anemia and tumor hypoxia [70]. One hypothesis is that anemia can increase hypoxic foci in GB tumors [71]. The hypoxic microenvironment of GB has been shown to be highly associated with tumor invasion and resistance to chemo- and radiotherapy, the main causes of death in GB patients [71,72]. Stüben et al. [73] showed that anemia reduced the efficacy of radiotherapy, and that its prevention by treatment with recombinant human erythropoietin partially restored the sensitivity of xenografted GB to fractionated irradiation.

As indicated above, NLR and dNLR were significantly correlated, but neither of these parameters was correlated with platelet or RBC counts in our selected cohort of GB patients. Interestingly, a score of 3, corresponding to the concurrent presence of high NLR or dNLR, high platelet, and low RBC counts for the preoperative blood test, was found to be an independent prognostic factor for shorter PFS and OS. Well-designed larger-scale studies are now required to test the efficacy of this score system for use as a clinical biomarker.

## 5. Limitations

This study has several limitations. First, this was a retrospective analysis with a small number of patients, which may be subject to several unavoidable biases. Second, only immunohistochemistry for IDH1-R132H was performed to assess the IDH status of the tumors. Sanger sequencing for IDH1/2 genes was not performed for all the cases. Third, we did not analyze the possible correlation with MGMT status, because this analysis is not mandatory for routine pathologic reports at our center. A review of the literature found no reports of correlations between NLR levels and MGMT promoter methylation status and showed that the prognostic role of NLR was not significantly modified by MGMT promoter methylation status [23,28]. Another limitation was the evaluation of hematological makers as categorical variables. The evaluation of these markers as continuous variables yielded no significant results in univariate analyses of PFS and OS (data not shown). Various statistical methods can be used for cutoff selection including, biomarker-oriented, and outcome-oriented approaches [74]. In this study, we used the maximally selected rank statistics from the ‘maxstat’ R package. This is an outcome-oriented method providing cutoff values corresponding to the most significant relationship to outcome. Outcome-oriented methods are generally expected to have better statistical indicators than biomarker-oriented methods [74]. In the future, a consensus should be clearly established, to determine the optimal statistical method for cutoff selection in this context. This would make it possible to make more meaningful comparisons between studies, and to define optimal cutoff values. The analysis of blood counts and their combined forms, without including other mediators of systemic inflammation such as acute-phase proteins including C-reactive protein and albumin, was also limiting. These proteins were not routinely analyzed at the time of patient admission to our medical center, suggesting that prospective studies are needed in this case. In addition to the analysis of these proteins, it would also be interesting to analyze the presence of circulating mesenchymal stem cells in the blood. These cells are capable of migrating toward GB and may be the source of GB-associated stromal cells (GASCs) [75]. Several studies have provided evidence that GASCs facilitate angiogenesis, invasion, and tumor growth [75]. Moreover, the percentage of GASCs in GB tumors is variable, high percentages being associated with a poorer OS [75].

## 6. Conclusions

We show here that cheap, routinely ordered preoperative blood tests for markers such as NLR, dNLR, RBC, and platelets can predict survival outcomes in patients with primary IDH-wildtype GB treated with the standard therapy. This approach could be used to develop personalized post-treatment monitoring based on closer clinical and radiological follow-up. Moreover, as high NLR and high dNLR are independent predictors of shorter PFS, both of these parameters could be useful as predictors of progression and resistance to the standard therapy. Data for hematological markers should be routinely recorded in clinical databases, such as the French glioblastoma biobank [76], at admission, before the administration of corticosteroids. This would make it possible to work with larger cohorts of IDH-wildtype GB patients, which could be stratified into different groups on the basis of sex, number of cycles of TMZ maintenance or survival, to identify and validate an optimal preoperative prognostic score based on NLR, dNLR, RBC, and platelet counts.

## Figures and Tables

**Table 1:** Characteristics of the patients with primary IDH-wildtype GB treated with the standard therapy. Abbreviations: dNLR, derived neutrophil-to-lymphocyte ratio; GTR, gross total resection (100%); KPS, Karnofsky performance score; LMR, lymphocyte-to-monocyte ratio; NLR, neutrophil-to-lymphocyte ratio; OS, overall survival; PFS, progression-free survival; PLR, platelet-to-lymphocyte ratio; PR, partial resection (<90%); RBC, red blood cells; SII, systemic immune-inflammation index; SIRI, systemic inflammation response index; STR, subtotal resection (≥90%); TMZ, temozolomide; WBC, white blood cells.

Patients		Number	%
**Patient characteristics**			
Age	median (range): 60 (36–81)		
● ≤60 years		43	51
● >60 years		42	49
Sex			
● Male		65	76
● Female		20	24
Preoperative KPS			
● ≤80%		14	16
● >80%		71	84
**Tumor location**			
Hemisphere			
● Left		42	49
● Right		43	51
Unilobar		46	54
● Frontal		16	19
● Temporal		12	14
● Parietal		13	15
● Occipital		4	5
● Limbic		1	1
Multilobar		39	46
**Extent of surgery**			
GTR		46	54
STR		26	31
PR		13	15
**Preoperative hematological markers**			
RBC	median (range): 4.79 (3.16–5.78)		
● ≤4.59 × 10^12^/L		23	27
● >4.59 × 10^12^/L		62	73
WBC	median (range): 8.66 (4.20–19.25)		
● ≤6.28 × 10^9^/L		9	11
● >6.28 × 10^9^/L		76	89
Neutrophils	median (range): 6.10 (2.03–16.56)		
● ≤3.68 × 10^9^/L		10	12
● >3.68 × 10^9^/L		75	88
Lymphocytes	median (range): 1.78 (0.40–5.51)		
● ≤1.31 × 10^9^/L		20	24
● >1.31 × 10^9^/L		65	76
Monocytes	median (range): 0.65 (0.06–1.22)		
● ≤0.37 × 10^9^/L		10	12
● >0.37 × 10^9^/L		75	88
Platelets	median (range): 237 (106–522)		
● ≤236 × 10^9^/L		41	48
● >236 × 10^9^/L		44	52
NLR	median (range): 3.18 (0.85–22.00)		
● ≤2.42		27	32
● >2.42		58	68
dNLR	median (range): 2.30 (0.69–12.29)		
● ≤1.89		32	38
● >1.89		53	62
LMR	median (range): 2.76 (0.83–24.54)		
● ≤2.06		24	28
● >2.06		61	72
PLR	median (range): 137.64 (51.08–645.00)		
● ≤180.90		62	73
● >180.90		23	27
SII	median (range): 772.13 (236.57–5715.87)		
● ≤502.39		23	27
● >502.39		62	73
SIRI	median (range): 1.95 (0.24–19.94)		
● ≤2.55		54	64
● >2.55		31	36
**Stupp regimen**			
Concurrent radiotherapy + TMZ		85	100
Adjuvant TMZ duration			
● ≤6 cycles		70	82
● >6 cycles		15	18
**Survival outcomes**			
Median PFS: 7.4 months (95% CI: [6.7; 8.7])			
Median OS: 17.7 months (95% CI: [14.5; 21.6])			

**Table 2:** Univariate Cox regression analysis of factors associated with PFS and OS. Abbreviations: Adj *p*, Bonferroni adjusted *p*-value; CI, confidence interval; dNLR, derived neutrophil-to-lymphocyte ratio; EOR, extent of resection; GTR, gross total resection (100%); KPS, Karnofsky performance score; LMR, lymphocyte-to-monocyte ratio; NLR, neutrophil-to-lymphocyte ratio; OR, odds ratio; PFS, progression-free survival; PLR, platelet-to-lymphocyte ratio; RBC, red blood cells; SII, systemic immune-inflammation index; SIRI, systemic inflammation response index; TMZ, temozolomide; WBC, white blood cells. * *p* < 0.05.

Univariate Analysis								
	PFS				OS			
Variables	OR	95% CI	*p*	Adj *p*	OR	95% CI	*p*	Adj *p*
Age (>60 years)	1.14	[0.74; 1.76]	0.556	1.000	1.32	[0.83; 2.09]	0.236	1.000
Sex (female)	0.87	[0.53; 1.45]	0.601	1.000	0.72	[0.42; 1.23]	0.232	1.000
KPS (>80%)	0.61	[0.34; 1.09]	0.097	1.000	0.45	[0.25; 0.82]	0.009 *	0.189
Hemisphere (left)	0.83	[0.54; 1.29]	0.410	1.000	0.85	[0.54; 1.34]	0.482	1.000
Lobe (multilobar)	1.38	[0.89; 2.15]	0.149	1.000	1.73	[1.08; 2.76]	0.022 *	0.462
EOR (GTR)	1.02	[0.65; 1.58]	0.945	1.000	0.99	[0.63; 1.57]	0.971	1.000
TMZ (>6 cycles)	0.13	[0.06; 0.29]	<0.001 *	<0.001 *	0.24	[0.12; 0.49]	<0.001 *	0.002 *
RBC (>4.59 × 10^12^/L)	0.58	[0.36; 0.95]	0.032 *	0.672	0.44	[0.26; 0.75]	0.002 *	0.042 *
WBC (>6.28 × 10^9^/L)	2.28	[1.09; 4.78]	0.029 *	0.609	3.89	[1.55; 9.77]	0.004 *	0.084
Neutrophils (>3.68 × 10^9^/L)	1.98	[0.98; 3.98]	0.056	1.000	3.80	[1.52; 9.50]	0.004 *	0.084
Lymphocytes (>1.31 × 10^9^/L)	1.43	[0.84; 2.43]	0.190	1.000	1.95	[1.09; 3.49]	0.025 *	1.000
Monocytes (>0.37 × 10^9^/L)	1.25	[0.64; 2.43]	0.519	1.000	1.94	[0.88; 4.26]	0.099	1.000
Platelets (>236 × 10^9^/L)	1.19	[0.76; 1.85]	0.444	1.000	1.61	[1.01; 2.57]	0.046 *	0.966
NLR (>2.42)	1.73	[1.06; 2.82]	0.027 *	0.567	2.11	[1.23; 3.61]	0.007 *	0.147
dNLR (>1.89)	2.12	[1.32; 3.39]	0.002 *	0.042 *	2.21	[1.32; 3.70]	0.002 *	0.042 *
LMR (>2.06)	0.64	[0.39; 1.06]	0.082	1.000	0.67	[0.41; 1.11]	0.122	1.000
PLR (>180.90)	0.92	[0.56; 1.50]	0.731	1.000	0.72	[0.43; 1.20]	0.208	1.000
SII (>502.39)	1.42	[0.86; 2.33]	0.170	1.000	1.83	[1.05; 3.21]	0.034 *	0.714
SIRI (>2.55)	1.62	[1.01; 2.59]	0.045 *	0.945	2.11	[1.29; 3.45]	0.003 *	0.063

**Table 3:** Multivariate Cox regression analyses of factors associated with PFS and OS. Abbreviations: CI, confidence interval; dNLR, derived neutrophil-to-lymphocyte ratio; dNLR-P-RBC, dNLR-platelet count-red blood cell count; KPS, Karnofsky performance score; NLR, neutrophil-to-lymphocyte ratio; NLR-P-RBC, NLR-platelet count-red blood cell count; OR, odds ratio; PFS, progression-free survival; RBC, red blood cells; TMZ, temozolomide. * *p* < 0.05.

Multivariate Analyses						
	PFS			OS		
Variables	OR	95% CI	*p*	OR	95% CI	*p*
**Multivariate analysis including NLR**						
Sex (female)	0.86	[0.50; 1.46]	0.571	0.52	[0.29; 0.93]	0.028 *
KPS (>80%)				0.83	[0.41; 1.66]	0.592
Lobe (multilobar)				1.28	[0.75; 2.20]	0.361
TMZ (>6 cycles)	0.12	[0.06; 0.27]	<0.001 *	0.26	[0.12; 0.54]	<0.001 *
RBC (>4.59 × 10^12^/L)	0.60	[0.36; 1.00]	0.048 *	0.42	[0.23; 0.75]	0.004 *
Platelets (>236 × 10^9^/L)				1.73	[1.06; 2.83]	0.030 *
NLR (>2.42)	2.02	[1.18; 3.44]	0.010 *	1.88	[1.06; 3.32]	0.030 *
**Multivariate analysis including dNLR**						
Sex (female)	0.79	[0.46; 1.34]	0.381	0.51	[0.28; 0.91]	0.023 *
KPS (>80%)				0.86	[0.43; 1.71]	0.665
Lobe (multilobar)				1.33	[0.78; 2.27]	0.294
TMZ (>6 cycles)	0.13	[0.06; 0.29]	<0.001 *	0.28	[0.13; 0.60]	<0.001 *
RBC (>4.59 × 10^12^/L)	0.62	[0.38; 1.02]	0.062	0.42	[0.23; 0.74]	0.003 *
Platelets (>236 × 10^9^/L)				1.67	[1.02; 2.73]	0.041 *
dNLR (>1.89)	2.28	[1.37; 3.79]	0.002 *	1.81	[1.05; 3.13]	0.033 *
**Multivariate analysis including NLR-P-RBC score**						
Sex (female)	0.83	[0.49; 1.40]	0.481	0.53	[0.30; 0.95]	0.034 *
KPS (>80%)				0.77	[0.39; 1.49]	0.435
Lobe (multilobar)				1.26	[0.74; 2.13]	0.397
TMZ (>6 cycles)	0.13	[0.06; 0.28]	<0.001 *	0.26	[0.12; 0.54]	<0.001 *
NLR-P-RBC score	1.51	[1.12; 2.02]	0.006 *	1.95	[1.42; 2.69]	<0.001 *
**Multivariate analysis including dNLR-P-RBC score**						
Sex (female)	0.79	[0.47; 1.33]	0.373	0.53	[0.30; 0.94]	0.030 *
KPS (>80%)				0.79	[0.41; 1.53]	0.480
Lobe (multilobar)				1.30	[0.76; 2.21]	0.333
TMZ (>6 cycles)	0.13	[0.06; 0.29]	<0.001 *	0.28	[0.14; 0.60]	<0.001 *
dNLR-P-RBC score	1.55	[1.17; 2.05]	0.002 *	1.90	[1.39; 2.60]	<0.001 *

## Data Availability

The data sets generated and/or analyzed in this study are available from the corresponding author under the authorization of the delegation for clinical research and innovation (DRCI, CHU, Angers).

## Supplementary Materials

The following are available online at https://www.mdpi.com/article/10.3390/cancers13225778/s1, Table S1: Relationship between hematological variables through Pearson’s chi-squared test.

## Abbreviations

AUC, area under the curve; CI, confidence interval; dNLR, derived neutrophil-to-lymphocyte ratio; dNLR-P-RBC, dNLR-platelet count-red blood cell count; EOR, extent of resection; GB, glioblastoma; GTR, gross total resection; IDH, isocitrate dehydrogenase; KPS, Karnofsky performance score; LMR, lymphocyte-to-monocyte ratio; MGMT, O6-methylguanine-DNA-methyltransferase; MPV, mean platelet volume; NET, neutrophil extracellular trap; NLR, neutrophil-to-lymphocyte ratio; NLR-P-RBC, NLR-platelet count-red blood cell count; OR, odds ratio; OS, overall survival; PLR, platelet-to-lymphocyte ratio; PFS, progression-free survival; PR, partial resection; RBC, red blood cells; RDW, RBC distribution width; ROC, receiver operating characteristic; SII, systemic immune-inflammation index; SIRI, systemic inflammation response index, STR, subtotal resection; TMZ, temozolomide; VEGF, vascular endothelial growth factor; WBC, white blood cells.
