# Supplementary material for: A Simple Preoperative Blood Count to Stratify Prognosis in Isocitrate Dehydrogenase-Wildtype Glioblastoma Patients Treated with Radiotherapy plus Concomitant and Adjuvant Temozolomide
Source: Cancers (Basel). 2021 Nov 18;13(22):5778. doi: 10.3390/cancers13225778 (PMC8616081; doi:10.3390/cancers13225778)
Supplement: Supplementary file 1 [file cancers-13-05778-s001.zip › cancers-1434407-supplementary.pdf]

A Simple Preoperative Blood Count to Stratify Prognosis in Isocitrate Dehydrogenase-Wildtype Glioblastoma Patients Treated with Radiotherapy Plus Concomitant and Adjuvant Temozolomide

Anne Clavreul, Jean-Michel Lemée, Gwénaëlle Soulard, Audrey Rousseau and Philippe Menei

Table S1. Relationship between hematological variables through Pearson's chi-squared test. Abbreviations: dNLR, derived neutrophil-to-lymphocyte ratio; LMR, lymphocyte-to-monocyte ratio; NLR, neutrophil-to-lymphocyte ratio; PLR, platelet-to-lymphocyte ratio; RBC, red blood cells; SII, systemic immune-inflammation index; SIRI, systemic inflammation response index; WBC, white blood cells.

	NLR		dNLR		Platelets		RBC	
	**Pearson's r**	***P***	**Pearson's r**	***P***	**Pearson's r**	***P***	**Pearson's r**	***P***
**RBC**	0.04	0.720	0.02	0.866	-0.22	0.046	1.00	/
**WBC**	0.26	0.017	0.36	< 0.001	0.20	0.062	-0.12	0.260
**Neutrophils**	0.38	< 0.001	0.39	< 0.001	0.23	0.033	-0.14	0.201
**Lymphocytes**	-0.20	0.067	-0.26	0.017	0.08	0.495	-0.15	0.169
**Monocytes**	-0.09	0.401	-0.13	0.225	0.16	0.146	-0.06	0.598
**Platelets**	0.05	0.654	0.08	0.489	1.00	/	-0.22	0.046
**NLR**	1.00	/	0.88	< 0.001	0.05	0.654	0.04	0.720
**dNLR**	0.88	< 0.001	1.00	/	0.08	0.489	0.02	0.866
**LMR**	-0.37	< 0.001	-0.38	< 0.001	-0.19	0.087	-0.03	0.792
**PLR**	0.30	0.005	0.36	< 0.001	0.22	0.046	0.07	0.507
**SII**	0.78	< 0.001	0.67	< 0.001	0.26	0.016	0.11	0.335
**SIRI**	0.52	< 0.001	0.49	< 0.001	0.10	0.385	-0.03	0.760
